# *Asplenium yishuiensis* (Aspleniaceae), a New Wintergreen and Medicinal Fern from Northern China, Achieves Freezing Tolerance via a Calcium-Mediated Osmotic Adjustment Pathway

**DOI:** 10.3390/plants15121773

**Published:** 2026-06-08

**Authors:** Jue Wang, Qingchun Wang, Ruohan Wang, Jian Wu, Zengli Liu, Wei Ma, Chaoyi Wang, Yuwei Fan

**Affiliations:** 1 Beijing Forestry University, Beijing 100083, China; wujian@bjfu.edu.cn; 2 School of Ecology and Nature Conservation, Beijing Forestry University, Beijing 100083, China; wangqch96@aliyun.com; 3 State Key Laboratory of Tree Genetics, College of Biological Sciences and Biotechnology, Beijing Forestry University, Beijing 100083, China; wangrh@bjfu.edu.cn; 4 Academy of Inventory and Planning, National Forestry and Grassland Administration, Beijing 100714, China; 13466579286@139.com (Z.L.); bmawei@163.com (W.M.); wangchaoyi@gjlcjghy.wecom.work (C.W.); fanyuwei2021@163.com (Y.F.)

**Keywords:** freezing tolerance, charge-driven anion accumulation, new species, medicinal, comparative anatomy, petiole vasculature, cell elongation, stress physiology

## Abstract

A long-standing paradigm in plant physiology proposes that cellular Ca^2+^ serves two primary functions: maintaining structural integrity and mediating intracellular signaling. Discovery of a morphologically similar congener of *Asplenium ruta-muraria* challenges this paradigm. The new species, *A. yishuiensis* from northern China, differs by its herbaceous to subsucculent and desiccation-sensitive laminae. Its mean mesophyll thickness is 232.8 μm, the number of vascular bundles at the petiole tips is 3–4, and the density of glandular hairs on the laminae averages 0.15 mm^2^/cm^2^. Conversely, *A. ruta-muraria* has a mesophyll thickness of 120–150 μm, two vascular bundles at the petiole tips, and glabrous laminae. Critically, *A. yishuiensis* lacks typical freezing adaptations common in temperate evergreens and does not exhibit poikilohydry. Its ability to remain evergreen at −20 °C reveals a previously unrecognized freezing tolerance mechanism. We hypothesize a two-tiered physiological strategy: (1) accumulation of Ca^2+^ complexes may indirectly lower cellular osmotic potential in some plants by promoting inorganic ions uptake; and (2) this mechanism varies among species, with *A. yishuiensis* using Ca^2+^ complexes more efficiently. Its total frond content of Ca + Mg reaches 12.66 g/kg dry weight, K reaches 22.7 g/kg, and V reaches 1.07 mg/kg. Specialized Ca^2+^-storage proteins and their accompanying ions likely enhance freezing tolerance in *A. yishuiensis*.

## 1. Introduction

The genus *Asplenium* (Aspleniaceae) is large, variable and subcosmopolitan. It comprises over 700 species, with the highest diversity in the tropics. China has about 90 species, including 17 endemics, and they are centered in tropical and subtropical regions [1]. Within this complex genus, *Asplenium ruta-muraria* is a widespread species. It is morphologically distinctive and circumboreal, native to Europe, East Asia, and eastern North America. *A. ruta-muraria* and its close relatives constitute the series *Asplenium* ser. *Variantia*, which is a natural taxonomic group within the genus *Asplenium*. However, members of this series exhibit considerable morphological plasticity and instability. Ching & Wu (1985) clarified the identity of *A. ruta-muraria* and confused species [2]. Species of *Asplenium* ser. *Variantia* exhibit diverse ecological adaptations. Understanding these adaptations provides insight into the evolution of stress tolerance mechanisms. This is a key question in plant biology.

*Asplenium* ser. *Variantia* has been subject to revision. A diploid entity was reported from dolomitic rocks in northern Italy. It has been described as *A. dolomiticum* [3]. Two Chinese taxa, *A. deqenense* and *A. suborbiculare*, were described in the 1960s. They have been treated as synonyms of *A. dolomiticum* by the Institute of Botany, Chinese Academy of Sciences. This is because they are interpreted as juvenile or depauperate forms of that species [4]. During recent botanical surveys in northern China, we discovered a population superficially resembling *A. ruta-muraria*. Detailed examination revealed consistent and distinct morphological and anatomical differences, which distinguish it from *A. ruta-muraria*. Here, we describe this population as a new species, *Asplenium yishuiensis*.

*Asplenium yishuiensis* Jue Wang, sp. nov.

**Type**—CHINA. Hebei: Baoding City, Yi County, Qianfo Hill scenic area, alt. ca. 450 m, 20 August 2025, *Jue Wang & Qing-Chun Wang* (holotype: BJFC).

**Diagnosis**—This species is similar to *Asplenium ruta-muraria*, but is readily distinguished by its herbaceous to subsucculent (vs. coriaceous to subcoriaceous) frond texture, sparsely glandular-hairy lamina surface (vs. glabrous), lack of a revivescent habit, and by having the vascular bundle at the distal end of the petiole divided into three or four (vs. two).

The discovery of *A. yishuiensis* not only contributes to taxonomic research, but also provides new physiological perspectives on stress adaptation. The fronds of *A. yishuiensis* lack the typical morphological and anatomical features for freeze tolerance common in temperate evergreen plants: a coriaceous texture; a dense indumentum (hairs, scales) or waxy/powdery coatings; narrow intercellular spaces and/or small, multi-layered mesophyll cells; low tissue water content; and thickened epidermal cell walls [5,6]. However, fronds of plants in their natural habitat remain fully vigorous at temperatures as low as −20 °C. Consequently, their overwintering survival likely depends on a unique suite of physiological and biochemical mechanisms. This suite is distinct from the poikilohydric (resurrection) strategy, which is commonly observed in desiccation-tolerant ferns.

According to Mitra (2015), plants absorb inorganic nutrients within specific limits, and excess is harmful [7]. Using X-ray fluorescence bioimaging and ICP (Inductively Coupled Plasma) analysis, we found that fronds of *A. yishuiensis* accumulate higher calcium (Ca) concentrations than those of *Nandina domestica*, which is the indicator plant of calcareous/calcium soil. Potassium (K) and chloride (Cl) concentrations in *A. yishuiensis* fronds are also much higher than those in *N. domestica*. The phosphorus (P) content in *A. yishuiensis* fronds approaches the high end of the range for plants [7] and far exceeds the mean phosphorus content of terrestrial plant leaves in China [8]. Moreover, its magnesium (Mg) content far exceeds the maximum uptake level of 0.4% of dry weight (DW) reported for crops ([7], Figure 3, Table 2). These support the hypothesis that *A. yishuiensis* relies on acids and inorganic ions to lower cellular osmotic potential and enhance freezing tolerance. This study investigates how this plant achieves osmotic adjustment through the accumulation of inorganic ions.

This study integrates classical taxonomy, comparative anatomy, and a novel physiological hypothesis. The hypothesis challenges a long-standing paradigm in the physiology of certain plant groups, particularly calcicole ferns. Historically, the recognized roles of cellular calcium have been confined to two canonical functions: structural requirements (e.g., cell wall stabilization, membrane integrity) and intracellular signal transduction (as a second messenger). Here, we propose a third dimension—calcium-mediated osmotic adjustment. This represents a previously overlooked function of calcium in plant stress adaptation.

## 2. Results

### 2.1. Description of Asplenium yishuiensis

Plants 3–6 cm tall. Rhizome scaly. Fronds caespitose, evergreen, monomorphic. Petioles 1–2 × lamina, green, adaxially sulcate with 1–2 deep grooves that become shallower when dry, evenly covered with short-glandular hairs. Rachis and lamina with similar hairs. Lamina herbaceous to subsucculent, ovate to broadly ovate-orbicular, 2–3-pinnate, lacking scales. Pinnae 3–7, in 1–3 alternate lateral pairs, pinnate to bipinnate, stalked. Basal pinnae largest, 7–22 mm long; upper pinnae are often ternate. Ultimate segments 3–7 × 2–5 mm, ovate-orbicular to broadly flabellate; apex rounded to obtuse, occasionally shallowly 1–3-lobed; margins shallowly serrulate or irregularly obtuse-toothed; base cuneate to broadly cuneate. Veins flabellately anadromous; veinlets reaching marginal teeth apices. Sori linear, borne along basiscopic veins. Indusia linear, light gray to brown, thinly membranous. Mature sporangia do not cover the entire segment surface.

**Distribution and habitat**—*Asplenium yishuiensis* is known only from a single locality in Qianfo Hill, where it grows in moist limestone crevices at ca. 430 m elevation.

**Figure 1:**
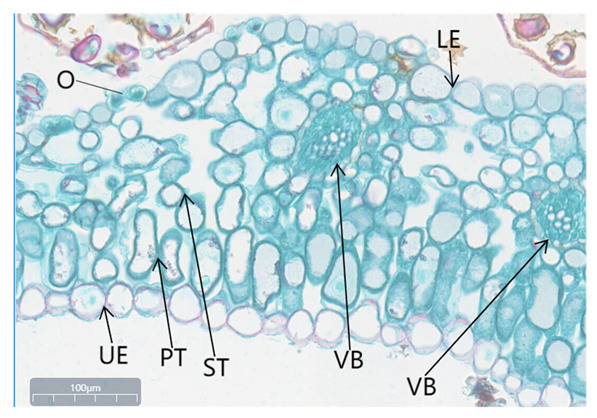
Pinnule anatomy of *Asplenium yishuiensis* in a partially dehydrated state (transverse section, 4 days after excision when stored in a sealed bag, stained with safranin and fast green). Abbreviations: LE, lower epidermis (cell diameter ca. 30 μm); O, ostiole; PT, palisade tissue; ST, spongy tissue; UE, upper epidermis (cell diameter ca. 30 μm); VB, vascular bundle.

**Notes**—Frond texture is strongly hydric-dependent. When fully hydrated, the lamina is turgid and succulent but lacks toughness. Upon mild dehydration, it becomes soft and flaccid with minimal shrinkage. It offers little tensile resistance and tears easily. When fully dry, the lamina becomes rather thin and flat. Venation is distinct in fresh and turgid leaves, especially abaxially, but obscure when dry. Petiolar sulcation and vascular bundles are also diagnostic. One or two grooves remain evident upon drying, though shallower. In *A. ruta-muraria*, the single groove is often flat when dry. Near the petiole-lamina junction, the petiole broadens distinctly and contains 3–4 vascular bundles. Additionally, the lamina lacks a distinct adaxial cuticle; adaxial and abaxial epidermal walls are not conspicuously thickened. The mesophyll comprises 3–4 cell layers. It consists of a single, relatively compact palisade layer and two to three layers of spongy tissue. The spongy tissue is composed of irregularly arranged cells with large intercellular spaces (Figure 1).

### 2.2. Quantitative Comparisons of Morphological and Anatomical Features

We have measured mesophyll thickness, vascular bundle number and density of glandular hair from 12 individuals. A total of 17 compound fronds were collected from 9 distinct individuals (one or two fronds per individual). Two transverse sections were prepared per compound frond, and two microscopic fields were measured per section, yielding two sets of statistical data, each comprising 34 independent measurements (68 measurements in total; Table 1). For the raw measurement data, please refer to the Supplementary File.

**Figure 2:**
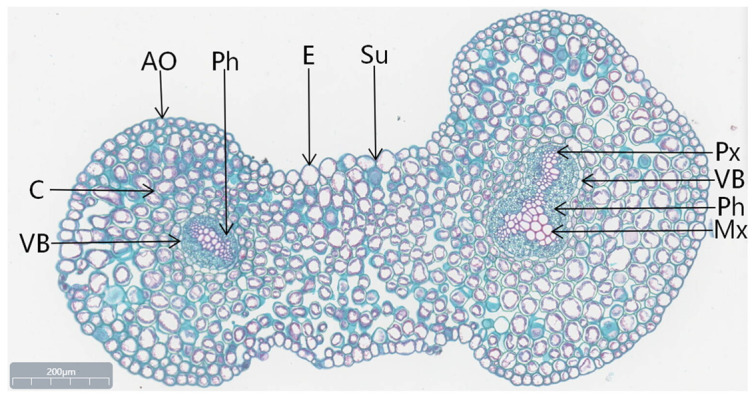
Petiole tip anatomy of *Asplenium yishuiensis* (transverse section stained with safranin and fast green), showing clear signs of ongoing bundle division. Abbreviations: AO, auriculate outgrowth; C, cortex; E, epidermis; Mx, metaxylem; Ph, phloem; Px, protoxylem; Su, sulcus; VB, vascular bundle.

We dissected petioles from twelve distinct individuals near the petiole-lamina junction. One petiole tip contained four vascular bundles, five petiole tips contained three vascular bundles, and the other six petiole tips contained only two vascular bundles but with clear signs of ongoing bundle division (Figure 2).

We randomly measured the density of glandular hair on laminae from nine distinct individuals (Table 2).

*A. ruta-muraria* does not occur in Beijing or the entire Hebei Province, so we were unable to collect its samples and therefore referred to literature data. Comparisons between *A. yishuiensis* and *A. ruta-muraria* revealed clear differences for all evaluated characters. In *A. yishuiensis*, the mean mesophyll thickness is 232.8 μm, the density of glandular hairs on the laminae averages 0.15 mm^2^/cm^2^ (Table 1 and Table 2), and the number of vascular bundles in the petiole tips is 3–4. Conversely, *A. ruta-muraria* has a mesophyll thickness of 120–150 μm, two vascular bundles at the petiole tips [9,10], and glabrous laminae [3]. These results confirm the species delimitation.

### 2.3. Quantitative Comparisons of Leaf Element Contents

**Figure 3:**
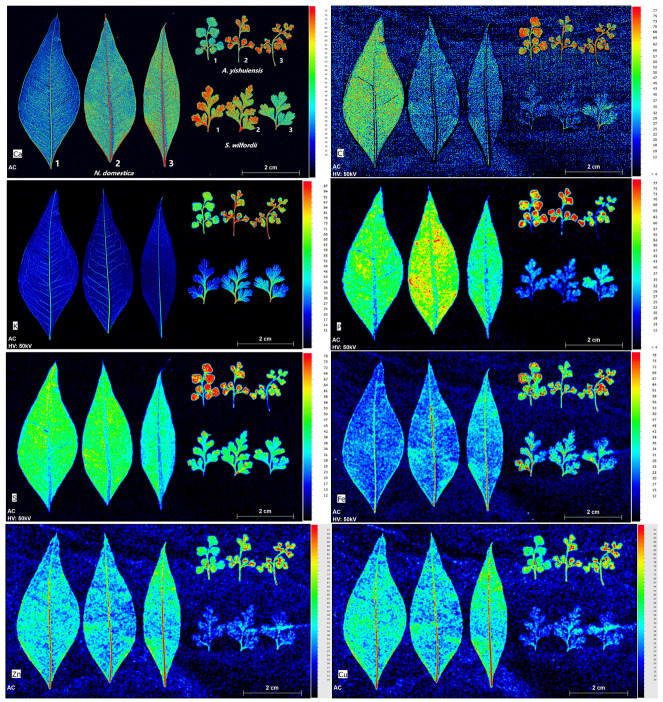
Bioimaging of Ca, Cl, K, P, S, Fe, Zn and Cu by X-ray fluorescence. The high-resolution distribution analysis of Ca, Cl, K, P, S, Fe, Zn and Cu in the leaves of three species (*Asplenium yishuiensis*, *Nandina domestica* and *Sitobolium wilfordii*) were analyzed using the X-ray fluorescence (XRF) spectrometer (M4 Tornado plus; Bruker, Berlin, Germany). A Lawrencium (Rh)-beam X-ray tube was equipped, and the working voltage and current were 50 kV and 600 μA, respectively. The leaves were scanned under vacuum (2 mbar) with a step distance of 60 μm and a single point scan time of 8 ms.

Using X-ray fluorescence bioimaging and ICP analysis, we compared the elemental concentrations in fronds of *A. yishuiensis* with those of the calcicolous indicator plant *Nandina domestica* and the widespread fern *Sitobolium wilfordii*. The results are shown in Figure 3 and Table 3.

#### 2.3.1. Elemental Accumulation in *A. yishuiensis* Fronds

The fronds of *A. yishuiensis* accumulate higher concentrations of Ca, iron (Fe), Mg, P, sulfur (S), zinc (Zn), and copper (Cu) than those of *N. domestica* and *S. wilfordii*. In X-ray fluorescence bioimaging, specimen *A. yishuiensis* 1 shows lower Ca and K signals than specimens 2 and 3. This discrepancy can be attributed to two factors. First, the fronds of specimen 1 are younger. Second, the prolonged slow drying (eight months) while pressed in a book likely led to both the loss of organic matter and the loss of elements, particularly K. Nitrogen (N) content was not directly measured. However, based on the strong positive and cross-species correlations between leaf nutrient pairs (e.g., Ca vs. Mg and N vs. P), it is inferred that the N concentration in *A. yishuiensis* fronds is also higher than that in *N. domestica* and *S. wilfordii*.

Limestone soils are typically poor in P and K; therefore, plants growing on such substrates would be expected to have lower P, K, and N contents than robust, cultivated *N. domestica*. Surprisingly, K and chloride (Cl) concentrations in *A. yishuiensis* fronds are remarkably higher than those in both *N. domestica* and *S. wilfordii*, a species that also inhabits limestone crevices. The P content in *A. yishuiensis* fronds reaches nearly 0.3% DW, which approaches the high end of the general plant range (0.05–0.5% DW [7]) and far exceeds the mean P content (0.146% DW) of terrestrial plant leaves in China [8].

Although the Ca content in *A. yishuiensis* is not necessarily much higher than that in *N. domestica*, the sum of Ca and Mg, reaching 12.66 g kg^−1^ DW, is substantially greater than the highest value (6.99 g kg^−1^ DW) in *N. domestica*. The Ca concentration is comparable to that of *S. wilfordii*, but the soluble Ca^2+^ concentration is likely higher. These soluble Ca^2+^ provide a large number of positive charges, and most Mg in plants is also soluble, with Mg^2+^ contributing additional positive charges. Furthermore, K exists as K^+^ in plant tissues, and the K^+^ content in *A. yishuiensis* fronds (22.7 g kg^−1^ DW) is three-fold or more than that in *N. domestica*, and similarly higher than in *S. wilfordii*. The high intracellular abundance of these cations must be balanced by corresponding anions. Consequently, the sum of N, P, S, and Cl concentrations in *A. yishuiensis* fronds is considerably higher than in *N. domestica* and *S. wilfordii*. Figure 3 and Table 3 demonstrate this, because these five elements are taken up predominantly as anions (e.g., Cl^−^, NO_3_^−^, H_2_PO_4_^−^, SO_4_^2−^). K^+^ and its accompanying anions form the foundation of osmotic potential in plant cells, and the roles of soluble Ca^2+^ and Mg^2+^ are not negligible. Even though the K content is 1.793 times the sum of Ca and Mg, both Ca^2+^ and Mg^2+^ are divalent cations, and each divalent cation attracts twice as many monovalent anions as K^+^.

Despite growing in the same limestone habitat, *A. yishuiensis* exhibits a much higher capacity for K^+^ uptake than *S. wilfordii*. Although exchangeable K^+^ in limestone is scarce, non-exchangeable potassium (NEK) is relatively abundant. Rhizosphere Ca^2+^ can promote the release of NEK from limestone [11]. This indicates that the rhizosphere Ca^2+^ concentration of *A. yishuiensis* far exceeds that of *S. wilfordii*. Rhizosphere Ca^2+^ concentration is closely related to plant Ca^2+^ concentration, and Mg^2+^ accumulation is also associated with Ca^2+^. These observations are consistent with our hypothesis of a Ca-mediated osmotic adjustment pathway, which contributes greatly to freezing tolerance.

#### 2.3.2. High Mg and Fe Contents Without Toxicity

The Mg content (7.69 g kg^−1^ DW) in *A. yishuiensis* fronds far exceeds the maximum uptake level of 0.4% DW reported for crops [7] and also surpasses 3.435 g kg^−1^ DW, which is the critical toxicity limit for tea plant leaves [7,12]. Nevertheless, no growth impairment is observed. Iron content is also high. It reaches 239 mg kg^−1^ DW without any signs of toxicity, approaching that in traditional iron-rich foods such as spinach and beef. Therefore, Fe^2+^ complexes extracted from *A. yishuiensis* might be explored as a treatment for iron-deficiency anemia.

#### 2.3.3. Vanadium Content and Medicinal Potential

Notably, *A. yishuiensis* exhibits vanadium (V) concentrations (1.07 mg kg^−1^ DW) remarkably exceeding the average value (0.502 mg kg^−1^ DW) reported for some medicinal plants [7]. V can act as an insulin-mimetic agent and can be used as a supplement in type 2 diabetes mellitus, along with other plants known to have hypoglycaemic effects. The V content in this plant remains far below the human toxic limit of 10 mg/day. Collectively, *A. yishuiensis* has potential as a valuable medicinal plant.

### 2.4. Conservation Status

Currently, fewer than 50 individuals are known, all from a single locality in the central mountainous area of a forest park. This area experiences high levels of tourist activity, which is detrimental to the conservation of the habitat. After the initial discovery, a second survey conducted eight months later revealed a sharp decline in the number of individuals, likely due to human disturbance and habitat degradation. Therefore, *Asplenium yishuiensis* should be assessed as “Critically Endangered” [CR B1c(i)+B2a+D] according to the IUCN Red List Categories and Criteria (IUCN, 2012). These criteria are a globally recognized system for evaluating the extinction risk of species. Given the specificity of its habitat, integral ecosystem conservation is essential for the protection of this endangered species.

## 3. Discussion

In Europe, *Asplenium ruta-muraria* often inhabits anthropogenic substrates such as walls, with populations exhibiting considerable variation in frond shape, size, and the degree of dissection [4,13]. Despite this pronounced phenotypic plasticity, the comparative analyses reveal clear differences between *A. yishuiensis* and *A. ruta-muraria* in key taxonomic characters, encompassing morphological, anatomical, and physiological traits.

### 3.1. Morphological Differentiation Between Asplenium ruta-muraria and Asplenium yishuiensis

#### 3.1.1. Frond Texture Differentiation: Coriaceous to Subcoriaceous vs. Herbaceous to Subsucculent

The fronds of *Asplenium ruta-muraria* are consistently described as coriaceous to subcoriaceous across a broad range of floristic treatments. Early European floras [14,15,16,17,18,19,20] established this characterization, which was later confirmed in regional treatments across Asia [21] and North America [22,23], as well as in modern European accounts [24,25,26,27,28]. Similarly, in recent online databases [29,30]. This textural uniformity contrasts with the pronounced phenotypic plasticity, underscoring the taxonomic stability of this trait.

**Figure 4:**
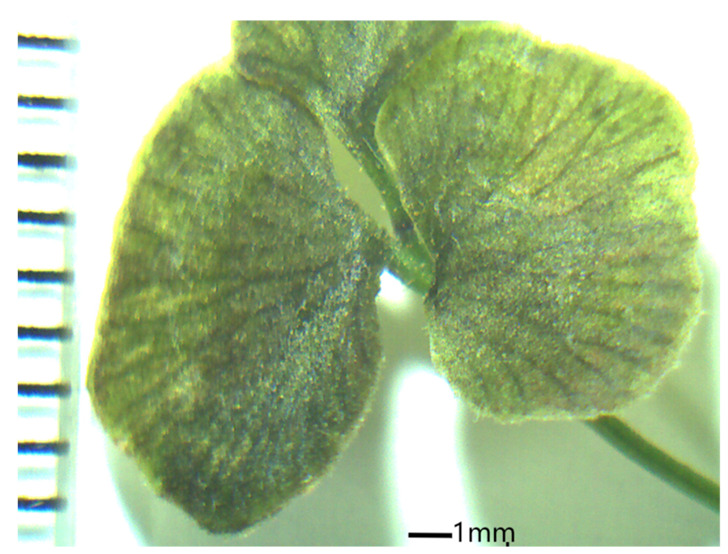
Stereomicrograph (surface view) of the leaf lamina of *Asplenium yishuiensis* after one day of excision and subsequent rehydration, showing the non-coriaceous texture.

*Yishuiensis’s* fronds differ markedly in texture. They are soft and conditionally subsucculent (Figure 4), appearing more herbaceous when dry. This visual assessment—suggesting a complete absence of a leathery texture—could lead to the interpretation of *A. yishuiensis* as a warm-adapted ecotype of *A. ruta-muraria*. Climatic data, however, do not support this hypothesis. *A. yishuiensis* is native to Yixian County in central Hebei Province, China, a region with a typical temperate monsoon climate characterized by cold, dry winters. Field records document severe winter conditions, with temperatures dropping to −20 °C in January 2026.

In contrast to the severe winters endured by *A. yishuiensis*, many documented localities of *A. ruta-muraria* in Europe experience relatively milder winter conditions [31]. In North America, a considerable portion of its range [22,32,33,34,35] lies within USDA Hardiness Zone 6b or warmer (average annual extreme minimum temperature > −20.6 °C). *A. yishuiensis* cannot be considered a warm-climate ecotype of the latter. The pronounced morphological differences between them are therefore more parsimoniously interpreted as species-level distinctions.

This interpretation is reinforced by the established diagnostic value of frond texture within the genus. Two closely related species pairs illustrate this pattern. *A. ruta-muraria* and *A. lepidum* are distinguished in part by a coriaceous texture versus thin, delicate, membranous fronds [3,17,18,19,27,36]. Similarly, *A. ruta-muraria* and *A. haussknechtii* differ by a coriaceous texture versus membranous, fragile, pellucid fronds [17,24,30]. The parallel contrast between the coriaceous *A. ruta-muraria* and the herbaceous to subsucculent *A. yishuiensis* thus provides strong comparative support for recognizing the latter as a distinct species.

#### 3.1.2. Frond Glandular Hair Differentiation: Sparsely Glandular-Hairy vs. Glabrous or Glandular Hairs Restricted to Petiole and Rachis

**Figure 5:**
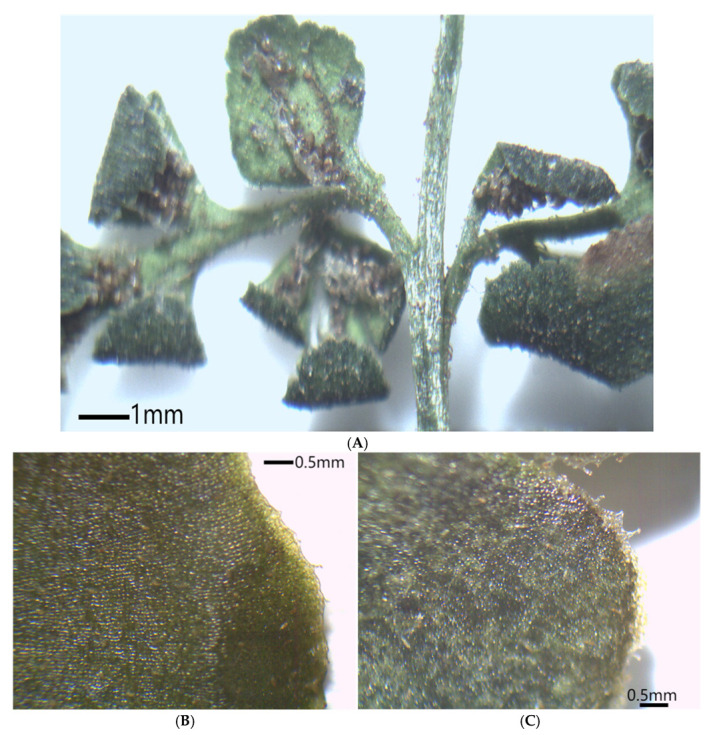
Stereomicrographs (surface view) of *Asplenium yishuiensis* under mild water deficit. (**A**) Overview of a pinna of a frond, showing the sparse short-glandular hairs on the petiole, rachis, and lamina surface (scale bar provided). (**B**,**C**) Details at higher magnification, each showing both the lamina margin and intercostal surfaces (scale bars provided). These images specifically document the clear morphology of the glandular hairs and their slightly higher frequency along the margin.

The lamina of *A. yishuiensis* entirely lacks scales, while the petiole, rachis, and lamina are sparsely covered with short-glandular hairs (Figure 5A–C). Because the extent and type of glandular hairiness are genetically determined [37], the laminar glandular hairs separate the new species from *A. ruta-muraria*.

The lamina of *A. ruta-muraria* is consistently described as glabrous in major floristic treatments, from Linnaeus (1753) to modern works such as Flora Europaea [3], Flora of North America [38], The Ferns of Britain and Ireland [25]. Even in descriptions of the two Chinese taxa once considered close to *A. ruta-muraria*—*A. deqenense* and *A. suborbiculare*—the authors did not report glandular hairs on the laminae, noting dense glandular hairs only on petioles and rachises as a core feature from *A. ruta-muraria* [2,21]. A detailed study by Muminov et al. further confirmed the absence of glandular trichomes on the lamina of *A. ruta-muraria*, noting only sparse, unicellular, non-glandular hairs [39].

The diagnostic value of glandular hairs is further illustrated by species closely related to *A. ruta-muraria*—*A. haussknechtii* and *A. lepidum*. Both possess a membranous lamina, resembling that of *A. ruta-muraria*. One of their primary distinguishing features is glandular pubescence: the former is predominantly glabrous to sparsely glandular-pubescent, whereas the latter bears short-stalked glandular hairs covering the entire plant [3,17,18,19,24,27,36]. Similarly, varieties such as *A. ruta-muraria* var. *brunfelsii* [17] and var. *schriesheimense* [37] are set apart from the typical variety in part by the presence of glands on the lamina. These examples reinforce the utility of glandular hairs in distinguishing *A. yishuiensis* from *A. ruta-muraria*.

### 3.2. Anatomical Differentiation Between Asplenium ruta-muraria and Asplenium yishuiensis

Comparative anatomy further underscores the distinctions between the two species.

#### 3.2.1. Petiole Anatomical Differentiation: Prominence of Auriculate Outgrowths and Number of Vascular Bundles

**Figure 6:**
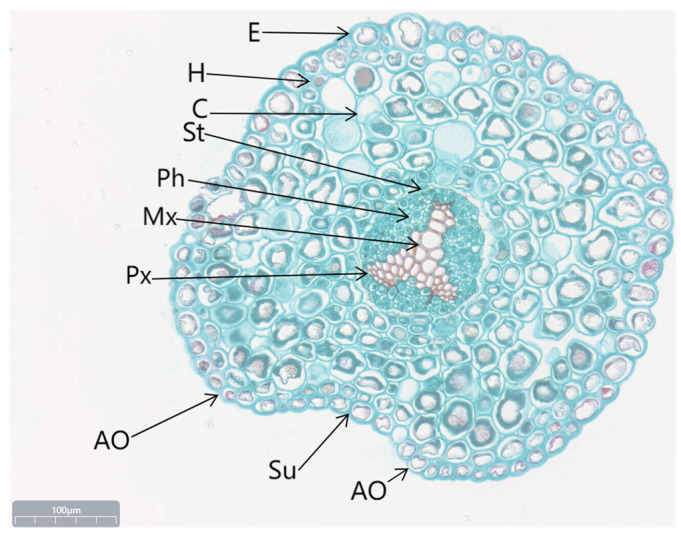
Sub-median petiole anatomy of *Asplenium yishuiensis* (transverse section stained with safranin and fast green). Abbreviations: AO, auriculate outgrowth; C, cortex; E, epidermis; H, hypodermis; Mx, metaxylem; Ph, phloem; Px, protoxylem; St, stele; Su, sulcus.

**Figure 7:**
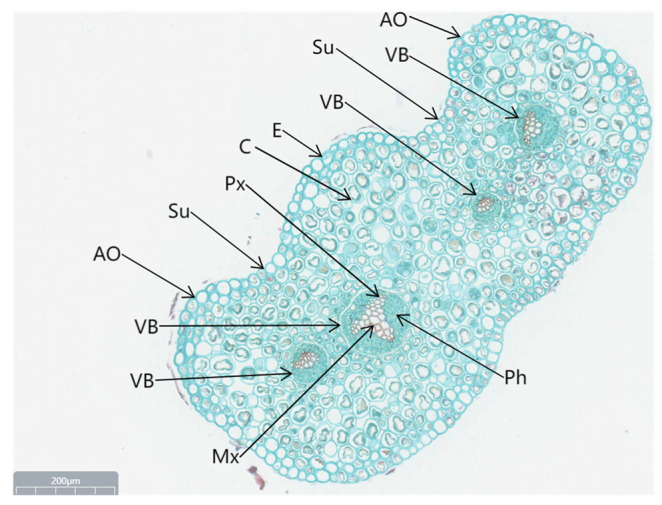
Petiole tip anatomy of *Asplenium yishuiensis* (transverse section stained with safranin and fast green). Abbreviations: AO, auriculate outgrowth; C, cortex; E, epidermis; Mx, metaxylem; Ph, phloem; Px, protoxylem; Su, sulcus; VB, vascular bundle.

A marked difference in petiole morphology is the development of auriculate outgrowths. At comparable levels in transverse section, these outgrowths are more prominent in *A. yishuiensis* than in *A. ruta-muraria*. In *A. yishuiensis*, weakly developed adaxial auricle-like outgrowths occur near the petiole base, becoming increasingly pronounced toward the lamina base, where they develop one or two distinct sulci (Figure 6). In contrast, the petiole of *A. ruta-muraria* is rounded at the base in transverse section; only at the lamina base does it bear similarly weak adaxial outgrowths, accompanied by small lateral depressions [10]. Moreover, the petiole near the petiole-lamina junction of *A. yishuiensis* broadens distinctly and contains three or four vascular bundles (Figure 7), whereas that of *A. ruta-muraria* shows little broadening and contains only two bundles [9,10].

The number of vascular bundles in the petiole is a relatively stable, genetically conserved trait [40] and serves as a key diagnostic character for generic and specific delimitation, particularly among closely related species [41,42,43,44]—aligning with the established view that the foliar and cauline vascular system provides the most reliable structural benchmark for comparative studies in fern systematics [45].

#### 3.2.2. Lamina Anatomical Differentiation: Cuticle Presence, Mesophyll Thickness, and Number of Cell Layers (3–4 vs. 4–5)

The foliar anatomy provides another key set of diagnostic characters. In *A. yishuiensis*, the anatomical features—lacking a distinct adaxial cuticle, with unthickened epidermal walls and a mesophyll of only 3–4 cell layers (Figure 1)—contrast markedly with those of *A. ruta-muraria*, which has a distinct adaxial cuticle, and 4–5 layers of more densely packed cells [10]. The mean mesophyll thickness of *A. yishuiensis* is 232.8 μm and is substantially greater than the typical thickness of *A. ruta-muraria* (120–150 μm, [10]). Correspondingly, the mesophyll cells and intercellular spaces are markedly larger in *A. yishuiensis*. These anatomical characteristics confirm that its fronds lack the coriaceous texture—a trait associated with tolerance to desiccation and freezing [5].

### 3.3. Winter Performance of Asplenium yishuiensis Fronds

**Figure 8:**
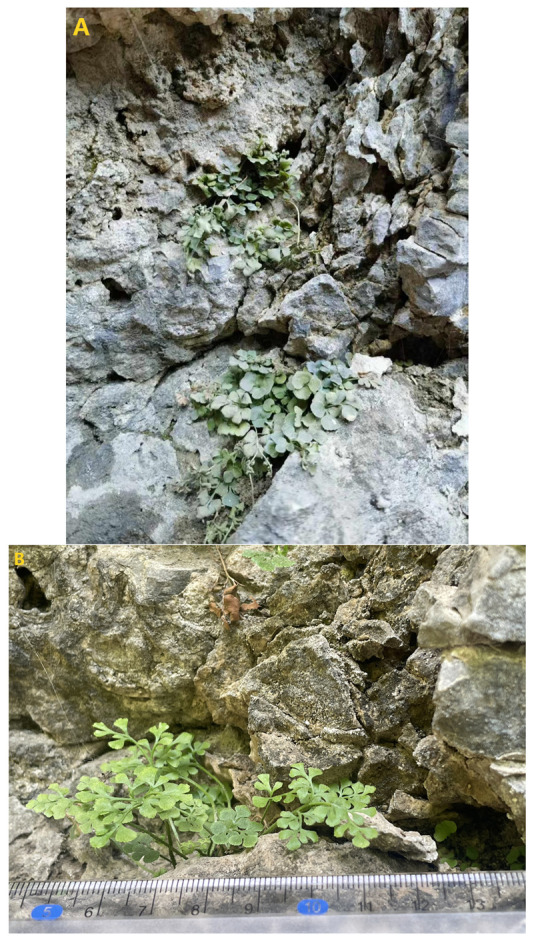
Community of *Asplenium yishuiensis* in its natural habitat. Qianfo Hill scenic area, Yi County, Hebei Province, China, 4 January 2026. (**A**) Note the evergreen fronds under an ambient temperature of −20 °C, 4 January 2026. (**B**) Provide scale bar, 5 May 2026.

The larger mesophyll cells and intercellular spaces in *A. yishuiensis* suggest higher tissue water content, which correlates with lower frost tolerance [46]. This, together with the absence of protective coverings and an anatomy that disfavors supercooling [47], would logically predict reduced freezing tolerance compared to *A. ruta-muraria*, which has been consistently documented as possessing an evergreen habit (e.g., [9,48,49,50,51,52,53,54]). Field observations, however, contradict this prediction. On 4 January 2026, *A. yishuiensis* in the Qianfo Hill remained evergreen despite temperatures dropping to −20 °C (Figure 8). In contrast, laboratory freezing experiments on *A. ruta-muraria* estimated its sporophyte survival at −10 °C [46], though its actual tolerance under natural conditions is likely lower than −10 °C.

Sometimes, fronds of *A. ruta-muraria* desiccate but persist without abscission, regreening upon rehydration in spring. This revival capacity defines desiccation tolerance (DT)—the ability to survive extreme water loss and recover normal function [55]—a phenomenon termed “resurrection” in vascular plants [56,57]. *A. ruta-muraria* tolerates desiccation for over a week, with relative water content dropping to 4–7%—far below the ca. 30% threshold for irreversible damage in most vascular plants [52,58]. Because the same cellular mechanisms underlying DT also protect against cellular dehydration caused by extracellular freezing [59], *A. ruta-muraria* exhibits strong poikilohydry during winter [10,60], which constitutes a primary physiological reason for its wintergreen habit.

DT ferns fall into two groups: (1) those with a well-developed cuticle, and (2) filmy ferns (mainly Hymenophyllaceae) with a simple frond structure and a rudimentary or absent cuticle [55]. *A. yishuiensis* lacks a distinct cuticle and has subsucculent fronds, fitting neither category. DT expression involves intricate protective pathways that safeguard cellular integrity against the mechanical and structural stress of dehydration stress [46,61]. Key anatomical adaptations include: (1) cell walls that shrink or fold without strain as the cytoplasm loses volume, preventing plasma membrane tearing—in contrast to the mesophyll collapse and enlarged intercellular spaces in desiccation-sensitive species; and (2) vacuole fragmentation into numerous smaller vacuoles, which increases cellular mechanical stability [62,63].

**Figure 9:**
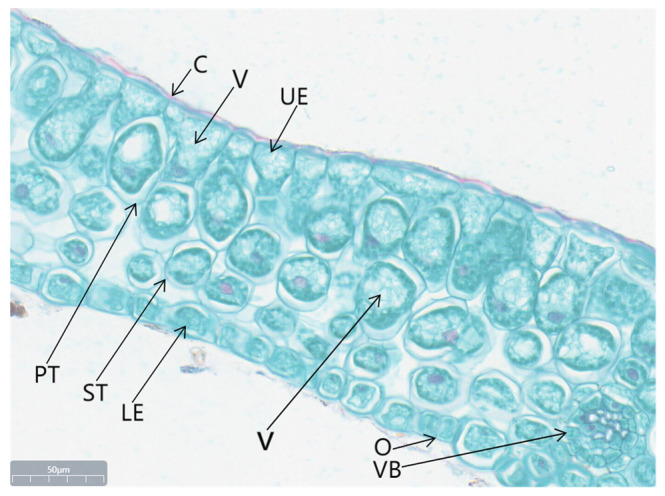
Pinnule anatomy of *Asplenium pekinense* in a partially dehydrated state (transverse section, 4 days after excision when stored in a sealed bag, stained with safranin and fast green). Abbreviations: C, cuticle; LE, lower epidermis; O, ostiole; PT, palisade tissue; ST, spongy tissue; UE, upper epidermis (cell exhibiting moderate shrinkage); V, vacuole (divided into numerous smaller compartments); VB, vascular bundle.

**Figure 10:**
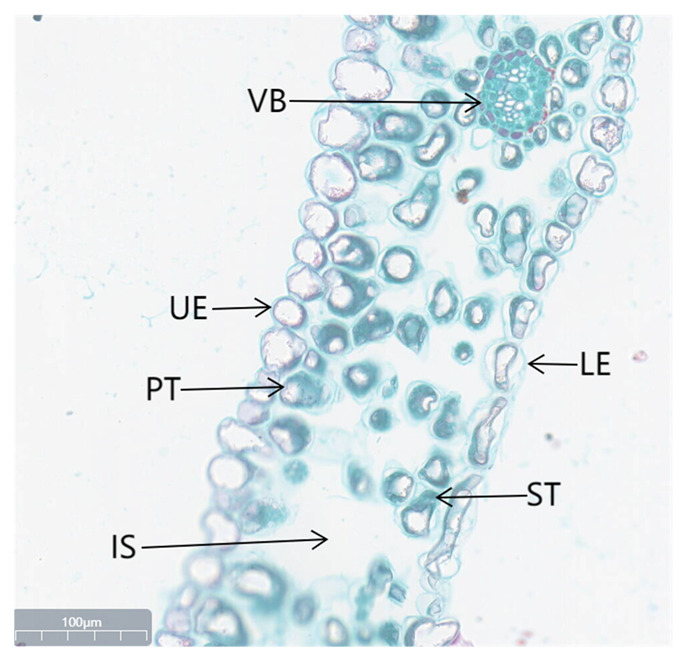
Pinnule anatomy of *Asplenium yishuiensis* in a partially dehydrated state (transverse section, 1 day after excision, stained with safranin and fast green). Abbreviations: IS, intercellular spaces; LE, lower epidermis; PT, palisade tissue; ST, spongy tissue; UE, upper epidermis; VB, vascular bundle.

In *A. pekinense*, pinnulae kept in a sealed bag for four days retained viability, showing a distinct cuticle, vacuole fragmentation, and limited epidermal shrinkage—indicating measurable DT (Figure 9). In stark contrast, *A. yishuiensis* pinnulae under identical conditions exhibited desiccation sensitivity (Figure 1 and Figure 10). Most cells lost considerable protoplasm; critically, all adaxial epidermal cells remained as empty cell walls, indicating irreversible cell death with no rehydration recovery. Unlike *A. ruta-muraria*, which survives desiccated for over a week, *A. yishuiensis* showed none of the key DT adaptations: cell walls did not shrink or fold, vacuole division was absent, and mesophyll collapse led to pronounced intercellular space enlargement (Figure 1 and Figure 10)—a hallmark of desiccation-sensitive plants. Meanwhile, the presence of a large vacuole implies that cells may have lost the ability to tolerate dehydration. All the above features confirm *A. yishuiensis* is desiccation-sensitive, and its wintergreen habit cannot be attributed to DT.

**Figure 11:**
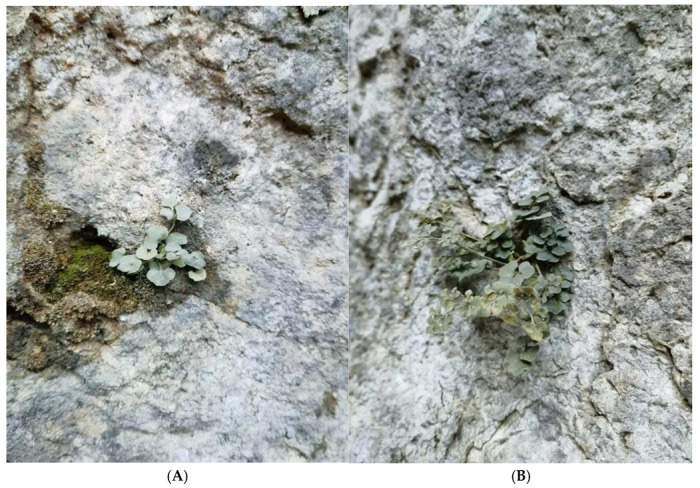
Winter habit of *Asplenium yishuiensis* in its natural habitat. Qianfo Hill scenic area, Yi County, Hebei Province, China, 4 January 2026. Note the evergreen fronds under an ambient temperature of −20 °C. (**A**) The upper portion of the plant diverges laterally from the crevice opening, placing it beyond the reach of most crevice-emitted airflow. (**B**) The crevice is shallow and wide—incapable of confining warm air even if present—and several fronds extend well away from it, fully exposed to ambient air at the same elevation.

Given that *A. yishuiensis* is desiccation-sensitive, its wintergreen fronds require a reliable source of unfrozen water under subzero temperatures. At the type locality in Qianfo Hill, several springs discharge continuously throughout winter, indicating geothermal activity. But substrate-derived heat cannot directly warm the fronds: leaf temperature is primarily determined by air temperature, solar radiation, wind, transpiration, and leaf traits, not by root-zone temperature—a well-established principle in plant physiology [64,65,66,67]. For substrate heat to affect the plant, a greenhouse-like crevice would be needed—sufficiently deep to envelop most of the plant, with a narrow opening to confine warm air. However, as shown in Figure 11, the spatial relationship between the plants and their crevices rules out any thermal effect from potential geothermal air currents. We therefore conclude that the wintergreen habit of *A. yishuiensis* is unrelated to geothermal heat, underscoring the novelty of its cold adaptation and the need for an alternative physiological explanation (Figure 11).

### 3.4. Two-Tiered Physiological Hypothesis for Wintergreen Fronds in Asplenium yishuiensis Under Severe Subzero Temperatures

Among vascular plants lacking typical freeze-tolerant morphology and DT mechanisms, *Chorispora bungeana* is a well-studied example. It exhibits strong inherent freezing tolerance and grows perennially in alpine environments. However, *C. bungeana* possesses several freeze-conducive anatomical features absent in *A. yishuiensis*: uniformly thickened epidermal walls, collenchyma around vascular bundles in the main vein, and 3–4 palisade layers [68]. Ecologically, its diminutive stature (3–10 cm) forms a prostrate rosette or cushion-like cluster readily covered by snow, providing insulation [69,70]. *A. yishuiensis*, by contrast, lacks such an insulating sheath. Multiple studies have demonstrated that *C. bungeana* possesses complex and efficient molecular mechanisms underlying its freezing tolerance [69,70,71,72,73,74,75,76,77]. Consequently, the winter-hardiness of *A. yishuiensis* appears remarkable and may rely heavily on unique physiological or biochemical adaptations.

K^+^ are universally recognized for establishing basal cell osmotic potential [78,79], but studies on *C. bungeana* attribute its freezing tolerance primarily to specialized metabolites—including antifreeze proteins (AFPs), soluble sugars, and free amino acids—that lower the freezing point, prevent dehydration via osmotic adjustment, control ice crystal formation, and stabilize membranes [70,76,77,80]. By analogy, *A. yishuiensis* must also accumulate cryoprotective solutes to achieve its observed freezing tolerance, but it is unlikely that these consist primarily of AFPs, free amino acids, or carbohydrates. Like *A. ruta-muraria*, *A. yishuiensis* grows on nutrient-poor limestone substrates with inherently limited photosynthetic productivity. Nevertheless, few data support the notion that ferns generally have lower foliar nutrient concentrations than angiosperms [81], suggesting greater photosynthetic economy. Given the high energetic cost of synthesizing organic osmolytes and especially AFPs [82], energetic economy would predict either the same freezing tolerance mechanism as its relative or a deciduous habit—neither of which occurs. This paradox suggests that the primary cryoprotective solutes in *A. yishuiensis* may be inorganic ions, which are far less costly to accumulate.

#### 3.4.1. Physiological Hypothesis One

The accumulation of Ca^2+^ complexes (e.g., bound to soluble Ca-binding proteins) in the cytosol and vacuole may indirectly lower cellular osmotic potential in some plants, especially calcicole ferns, by promoting ion uptake, thereby improving freezing tolerance.

##### Use of Alternative Inorganic Ions (e.g., Na^+^) for Vacuolar Osmotic Adjustment

K^+^ is a highly effective osmoregulatory ion due to its availability, mobility, and low molecular weight, yet sufficient K^+^ for both growth and osmotic adjustment is not always available under stress. Some plants therefore employ alternative inorganic ions [78]. Halophytes, for example, sequester large quantities of inorganic ions, particularly Na^+^, into the vacuole, lowering its osmotic potential to maintain water uptake under saline stress, while balancing cytoplasmic osmotic pressure with a relatively small amount of compatible organic solutes. This strategy—relying primarily on inorganic ions for vacuolar osmotic adjustment—substantially reduces the investment of photosynthates in energy-intensive organic osmolytes [82,83,84].

However, excess Na^+^ is generally highly toxic to most plants: Na^+^ accumulation in leaves often occurs at the expense of Ca^2+^ and K^+^; a strong negative correlation exists between mesophyll Na^+^ and photosynthetic capacity [85], raising the question of how halophytes sequester Na^+^ into vacuoles without disrupting organellar function; and Na^+^ disrupts plasma membrane integrity [86], yet halophytes must maintain tonoplast integrity to prevent passive Na^+^ efflux from the vacuole back into the cytoplasm—a requirement whose mechanistic basis remains unresolved, limiting translation to crop salt tolerance strategies. Moreover, halophytes still require compatible solutes (e.g., proline, glycine betaine) to balance cytoplasmic osmotic pressure—an additional energetic cost that may limit applicability to crops.

##### Ca^2+^ Indirectly Lowers Cellular Osmotic Potential

While calcium itself has been recognized as an osmolyte, free Ca^2+^ itself contributes negligibly to osmotic potential [87]. The mechanism by which Ca^2+^ indirectly contributes to lowering cellular osmotic potential in some plants proposes a previously overlooked third dimension of Ca function beyond its canonical structural and signaling roles.

##### Substantial Ca Accumulation Is Not Restricted to Calcicole Plants

Ca is the third most abundant nutrient in soils and is essential as Ca^2+^ [88]. It can accumulate substantially in plant tissues, not only in calcicoles—e.g., in potato tubers after foliar application [88], in wild-type and cold-acclimated *Chorispora bungeana* [73], and in alpine scree species compared to low-elevation plants [70]. Moreover, vacuolar total Ca can increase from ca. 20 to 100 mmol L^−1^ in K-deficient barley shoots [85], suggesting a role in charge and/or osmotic balance. Collectively, these observations indicate that Ca^2+^ uptake is not strictly metabolically regulated but is influenced by substrate concentration and root xylem permeability [89,90].

Most accumulated Ca^2+^ in plant cells is sequestered into the vacuole because cytosolic free Ca^2+^ ([Ca^2+^]cyt) must be maintained at a resting level of approximately 100–200 nmol L^−1^. This strict regulation is essential, as [Ca^2+^]cyt serves as a second messenger: upon stress stimuli (e.g., low temperature, drought, salinity), [Ca^2+^]cyt transiently increases and reaches a certain threshold, triggering a cascade of physiological and biochemical responses to counteract the stress [70,86,91]. Thus, the cytosol functions as a Ca^2+^ signaling space, requiring nanomolar [Ca^2+^]cyt to ensure undisturbed metabolism in unstimulated cells and to provide the dynamic range necessary for transient Ca^2+^ elevations [91,92].

The vacuole is the primary intracellular Ca^2+^ storage compartment, with total Ca^2+^ concentrations (free + bound) reaching up to 80 mmol L^−1^ [85,93]. However, most of this Ca^2+^ is tightly bound; under vacuolar acidic conditions, free Ca^2+^ is limited (0.1–5.0 mmol L^−1^; [85,87,92,93,94]) and tends to precipitate as calcium oxalate (CaOx; [95,96]). Even at the upper end of this range, free Ca^2+^ contributes negligibly to vacuolar osmotic potential. Even if all 80 mmol L^−1^ of vacuolar Ca^2+^ were osmotically active, the resulting potential would be at most −0.2 MPa—far below total cellular osmotic potential (−1.0 to −2.5 MPa). Thus, vacuolar Ca^2+^ makes only a relatively minor contribution to cellular osmotic adjustment, especially given that much of the 80 mmol L^−1^ vacuolar Ca^2+^ is sequestered as CaOx. Nevertheless, it has been suggested that Ca^2+^ can maintain osmotic stabilization through active transmembrane transport [72], demonstrating that Ca^2+^ accumulation is associated with plant stress resistance.

##### Association Between Ca^2+^ Accumulation and Plant Stress Resistance

Multiple studies have linked Ca^2+^ accumulation to plant stress resistance. In particular, cell types of *Arabidopsis thaliana*, vacuolar K^+^ accumulation may compensate for reduced vacuolar Ca^2+^ through an unknown mechanism, suggesting an interdependence between the two elements in osmotic, ionic, and charge balance [79,85,97]. Similarly, in the alpine subnival plant *Chorispora bungeana*, Ca^2+^ accumulates preferentially in tissues with greater cold hardiness [69,70,71,72]. Low-temperature stress induces a significant increase in vacuolar Ca^2+^ content of regenerated seedlings, and elevated free vacuolar Ca^2+^ has been proposed to increase solute concentration, thereby preventing intracellular freezing and reducing stress injury [72,75]. Sun (2011) therefore suggested that high Ca^2+^ concentrations may be essential for the normal growth of alpine scree plants under extreme conditions [70]. More broadly, an optimal Ca^2+^ supply also effectively enables plants to thrive under water deficit [98].

##### The Apparent Paradox Between Ca^2+^ as an Osmoticum and the Narrow Concentration Range of Free Ca^2+^ in Plant Points to a Subtler, Easily Overlooked Biochemical Principle

Although the freezing tolerance of *C. bungeana* relies primarily on AFPs and organic osmolytes, if Ca^2+^ accumulation contributed negligibly to vacuolar osmotic potential, the conclusions of the cited studies would be called into question. However, the concentration range of free Ca^2+^ in plants is generally narrow and the upper range of vacuolar free Ca^2+^ (ca. 5.0 mmol L^−1^) likely applies specifically to calcicole species. This apparent paradox points to a subtler biochemical principle that is easily overlooked: the entry of Ca^2+^ into cells requires equivalent accumulation of anions to maintain charge balance. Because Ca^2+^ is divalent, it requires twice the number of monovalent anions; thus, 5.0 mmol L^−1^ of free Ca^2+^, accompanied by its charge-balancing anions, effectively represents 15.0 mmol L^−1^ of osmotically active particles.

Second, after large quantities of Ca^2+^ enter the vacuole, only a small fraction (<5.0 mmol L^−1^) remains free; the remainder, though chelated by organic acids or proteins, still contributes to the cell’s overall charge-balancing requirement, necessitating a corresponding increase in accompanying anions. Moreover, certain organic solutes may chelate Ca^2+^ through precisely coordinated oxygen atoms rather than via organic anions, further complicating the charge balance dynamics. Plants can synthesize organic anionic groups, but this process, like the alternative strategy of exporting excess Ca^2+^ from the cell, is energetically costly [99]. This charge balance can be achieved by inorganic anions [100]. Hoagland and Davis (1923) determined that 80–90% of positive ions in plant cells are balanced by inorganic anions, with the remainder plausibly ascribed to organic anions such as oxalate [101]. Consistently, Kour et al. (2023) noted that Ca^2+^ maintains vacuolar anion concentrations [98]. Moreover, decreases in extracellular osmotic potential and increases in intracellular Ca^2+^ concentration may activate anion channels [102], facilitating anion uptake. Thus, while free Ca^2+^ itself contributes negligibly to osmotic potential [87], the charge-balancing inorganic anions attracted by soluble Ca^2+^ can double or triple its osmotic impact—at minimal energetic cost, making calcium-binding proteins/chelators indirect but powerful osmotic regulators.

Calcium-binding proteins (CaBPs) are not confined to the vacuole—e.g., located on vacuolar membranes or within the lumen of vacuoles [93,103]; they are also present in other subcellular compartments—e.g., calreticulin, a major Ca^2+^-storage protein in the endoplasmic reticulum (ER), binds 20–50 moles of Ca^2+^ per mole [85,92,93,104,105]. Although such a soluble protein contributes only a single osmotically active particle regardless of how many Ca^2+^ it binds, the charge-balancing requirement for the bound Ca^2+^ demands the accumulation of a proportional number of accompanying anions. Thus, this mechanism suggests that anion channels are involved in osmotic adjustment in both vacuole and cytosol [102]. This indirect osmotic role of Ca^2+^—mediated by its charge rather than its concentration—has been largely overlooked in plant physiology. However, to maintain nanomolar [Ca^2+^]cyt, the cells of most plants are unlikely to contain large quantities of Ca^2+^-storage proteins in cytoplasm; if such Ca^2+^-storage proteins had lower Ca^2+^ affinity than calcium sensor proteins, leakage of free Ca^2+^ from these buffers would interfere with the fidelity of [Ca^2+^]cyt signaling.

##### How Do Certain Plants Achieve High Rates of Ca^2+^ Uptake While Maintaining Cytosolic Free Ca^2+^ Homeostasis and Preserving Its Second Messenger Function?

As noted above, under stress conditions, [Ca^2+^]cyt transiently increases to fulfill its signaling function, but must then be rapidly restored to nanomolar levels to prevent disruption of cellular metabolism and cell death [72,91]. This dynamic regulation is achieved by Ca^2+^-ATPases, Ca^2+^ channels, and cation exchangers (CAX) located on plasma and organellar membranes, which pump excess [Ca^2+^]cyt out of the cell or into storage organelles such as the vacuole [72,73,106]. In stress-tolerant plants—such as K-deficient barley shoots, in which vacuolar total Ca reaches 100 mmol L^−1^—Ca^2+^-regulatory proteins may be upregulated to rapidly transport Ca^2+^ out of the cytosol. Similarly, to alleviate continuous calcium oxalate precipitation and allow more time for Ca^2+^ transport, other CaBPs may also be upregulated, increasing soluble Ca^2+^ content. Consequently, vacuolar Ca^2+^ sequestration is a characteristic feature of cold-tolerant plants under low-temperature stress. In contrast, non-cold-tolerant plants exhibit large amounts of Ca^2+^ in the cytoplasm under such conditions, leading to cell death, presumably due to their inability to upregulate sufficient Ca^2+^-regulatory proteins [72].

The upregulated CaBPs in stress-tolerant plants discussed above are inducible rather than constitutive, supported not only by the observation that Ca^2+^ content increases in regenerated plantings under chilling induction and decreases under favorable conditions [71,72], but also by the following experimental evidence: potato transgenic lines overexpressing CAX1s exhibited increased vacuolar Ca^2+^ accumulation and CaOx crystals in leaf cells under 1 mmol L^−1^ Ca treatment—crystals absent in wild-type [96]; SsCAX1-overexpressing *Arabidopsis thaliana* showed no growth difference under normal conditions but had markedly higher shoot Ca^2+^ content than wild-type [86]; in wild *C. bungeana*, Ca^2+^ content was highest in leaves, intermediate in petioles, and lowest in roots [71,75], reflecting that exposed organs require more Ca^2+^-regulatory proteins [72]; four CML-encoding genes were significantly upregulated in stems of *Dendrobium officinale* under high-Ca conditions (AR; [107]); and in *cax1 mutants facing higher apoplastic Ca^2+^ ([Ca^2+^]apo) and reduced mesophyll Ca^2+^ secretion, vacuolar Ca^2+^-ATPase activity increased by 36% compared to wild-type Columbia-0, correlating with greater abundance of tonoplast-localized ACA4 and ACA11 [97].

Not all plants can upregulate Ca-regulatory proteins under stress. Moreover, this Ca-mediated osmotic adjustment pathway—which circumvents organic solute synthesis—is also energetically costly, and its contribution to freezing tolerance in *C. bungeana* requires further investigation. Furthermore, even if cytosolic CaBPs could be concurrently upregulated, their extent would likely be limited by the need to preserve [Ca^2+^]cyt signaling. Excess Ca storage leading to CaOx crystal formation is therefore primarily observed in calcicole plants.

Wild *C. bungeana* enhances osmotic adjustment by accumulating both organic osmolytes and inorganic ions—a dual strategy enabling it to survive harsh environments [74], and involving CaBPs and their accompanying anions in the cytosol as a sophisticated layer of this adaptive mechanism. We may now examine the application of this Ca-mediated osmotic adjustment pathway in calcicole ferns.

##### The Osmotic Role of Soluble CaBPs in Calcicole Ferns Under Chronic AR Stress

The osmotic role of soluble CaBPs—often overlooked due to their low abundance in model plants—merits attention in calcicole ferns, where, under chronic AR stress, their synthesis/accumulation may attract more inorganic anions to lower cellular osmotic potential more effectively than in non-calcicole plants.

Given that cell-specific storage of Ca^2+^ (not limited to the vacuole) is essential in plants to regulate [Ca^2+^]apo; [97], it follows that soluble CaBPs within the cell participate in maintaining a dynamic balance between intracellular and extracellular Ca content. This inference necessitates that calcicole ferns possess more CaBPs than those in non-calcicole plants, or possess CaBPs/chelators with higher Ca^2+^ affinity. Importantly, these CaBPs/chelators in calcicole ferns under chronic AR stress are constitutive rather than inducible.

##### Regulation of Total Ca^2+^ Uptake by Transmembrane Ca^2+^ Equilibrium in Calcicole Ferns

In contrast to stress-tolerant plants, calcicole ferns have high constitutive Ca^2+^ levels even under normal conditions. Hoagland and Davis demonstrated that cell sap ion concentrations are much higher than in the external medium and can be increased by raising external concentrations [101]. Calcicole plants continuously secrete organic acids to solubilize immobilized ions from limestone, enriching their rhizosphere in Ca^2+^. Under such steep concentration gradients, passive Ca^2+^ influx becomes substantial—plants cannot completely “close the valve”. Thus, calcicole plants inevitably take up large quantities of Ca^2+^ passively.

However, obligate calcicole plants are not necessarily all Ca-loving or Ca-dependent; some are low-Ca species (e.g., *Adiantum capillus-veneris*). Calcicole ferns generally have higher Ca, Mg, and N concentrations than low-Ca species [81,99]. If they were merely passive Ca^2+^ accumulators, then: (i) transplanting to balanced soils should reduce tissue Ca without compromising vitality—yet *A. ruta-muraria* failed to survive when calcareous mortar was insufficient [108] and is considered an absolute calcicole [109] (p. 164); (ii) based on selective ion uptake of plants, chronic Ca stress would not require upregulation of Ca^2+^ transporters—ordinary transporters would suffice, and only continuous oxalate synthesis would be needed until crystals clogged cellular functions, but no such reports exist; (iii) Species with a high Ca demand (e.g., *Nephrolepis auriculata*) can meet their requirement by mobilizing Ca from acidic soils, making its availability in the rhizosphere actually high—indicating active regulation rather than passive accumulation [110]. Therefore, under chronic AR stress, the substantial Ca^2+^ uptake by calcicole ferns is not entirely passive; rather, most excess Ca^2+^ is actively absorbed, or a dynamic influx-efflux equilibrium is maintained. This is supported by Wang et al. (2011), who showed that in limestone environments, *Parathelypteris glanduligera* and *Pteris cretica* var. *nervosa* maintain Ca^2+^ within an adaptable range by restricting uptake [110].

Franceschi (1989) demonstrated that CaOx crystal formation is reversible: recently formed crystal bundles dissolved within about 3 h [89]. Meanwhile, Oxalate synthesis and degradation in leaf tissues are genetically regulated and occur in parallel [89,90]. Under Ca^2+^ deficiency induced by development or intense growth, CaOx crystals are degraded via oxalate-degrading enzymes (e.g., oxalate oxidase), indicating their remobilization potential, although older crystals are more resistant. This reversible equilibrium between crystallization and solubilization dispels the idea that CaOx formation is a dead-end process and may explain why crystal abundance correlates with plant Ca^2+^ levels and soil Ca^2+^ availability [90]. In calcicole ferns, whose vacuoles contain abundant CaOx crystals, total Ca^2+^ uptake must be regulated by this equilibrium, as shown by observations that CaOx crystal form, size, and quantity vary with environmental Ca^2+^ concentration, thereby reducing [Ca^2+^]apo, and that cell-specific Ca^2+^ storage is essential for regulating [Ca^2+^]apo [97,110].

##### A Robust Ca^2+^-Buffering Mechanism in Calcicole Ferns—Likely Involving Specialized Soluble CaBPs/Chelators in Both Vacuole and Cytosol—Is Required to Maintain Intracellular-Extracellular Ca^2+^ Homeostasis Under Chronic AR Stress

Given that CaOx modulates Ca^2+^ influx in calcicole ferns, Ca^2+^-regulatory proteins (transporters) and other CaBPs involved in Ca^2+^ efflux and recovery [98] must play an antagonistic role under AR stress, preventing uncontrolled Ca^2+^ entry. Plasma Membrane Ca^2+^-ATPase plays a central role in cytosolic and extracellular Ca^2+^ homeostasis [73], but its function likely extends beyond simply pumping Ca^2+^ out of the cytosol. To enable metabolite-bound Ca^2+^ to participate in maintaining a dynamic balance between intracellular and extracellular Ca^2+^ content under chronic AR stress, calcicole ferns must possess a robust Ca^2+^-buffering mechanism. This may involve constitutively more abundant or more active Ca^2+^ transporters and CaBPs than those in non-calcicole plants, or—more likely—the evolution of specialized Ca-buffering proteins or chelators that are soluble and distributed in both the vacuole and cytosol. Examples may include organic chelators with precisely coordinated oxygen atoms, or soluble proteins rich in acidic amino acids—which contain other non-acidic residues and have a near-zero net overall charge; both combine high Ca^2+^-binding capacity. By sequestering large amounts of Ca^2+^ in a chemically “occupied” form, these molecules serve as Ca^2+^ buffers and, together with their accompanying inorganic anions, as genuine osmolytes—preventing free Ca^2+^ toxicity while lowering the electrochemical/concentration gradient for Ca^2+^ influx under AR stress.

Although the extent to which constitutive Ca^2+^ complexes in calcicole ferns contribute to vacuolar and cytosolic osmotic adjustment requires further investigation, their contribution is likely greater than in non-calcicole plants. Calcicole ferns typically grow on nutrient-poor limestone substrates with inherently limited productivity. Rather than expending energy on organic osmolyte synthesis, it would be energetically favorable for these plants to absorb inorganic anions from the environment to the greatest extent possible. Limestone habitats are rich in Ca, and specialized chelators may bind more Ca^2+^ per molecule than ordinary CaBPs. The mechanisms that efficiently utilize Ca^2+^ to drive anion uptake carry significant ecological adaptive value. The accumulation of large quantities of soluble Ca^2+^ with its accompanying inorganic anions may lower vacuolar osmotic potential more effectively in calcicole ferns than in non-calcicole plants. Additionally, specialized cytosolic chelators may represent a “cheap storage” strategy: retaining Ca^2+^ and its accompanying anions as osmotic contributors while avoiding the energetic cost of constant efflux and the signaling disruption caused by elevated free Ca^2+^.

##### Specialized Ca-Buffering Proteins or Chelators in the Cytosol of Calcicole Ferns Would Not Interfere with [Ca^2+^]cyt Signaling

Why would specialized Ca-buffering proteins or chelators in the cytosol of calcicole ferns not interfere with [Ca^2+^]cyt signaling? The inference derives from the failure of *A. ruta-muraria* to survive in lime-deficient soil [108], suggesting that such specialized buffers may be constitutively positioned at Ca^2+^ entry points of plasmalemma, sequestering Ca^2+^ before signaling proteins (e.g., calmodulin; [107]) can bind it. If these chelators have higher Ca^2+^ affinity than other CaBPs, they could deprive the plant of metabolically necessary Ca^2+^ without AR stress—explaining why SsCAX1-overexpressing *A. thaliana* showed Ca^2+^ deficiency on Ca^2+^-free medium [86].

Additional support comes from other Ca^2+^-storage compartments: calreticulin buffers [Ca^2+^]cyt [85,94], and its overexpression increases ER Ca^2+^-storage without impairing growth [92,105]; chloroplasts store Ca^2+^ up to 15 mmol L^−1^ yet maintain stromal free Ca^2+^ at ~150 nmol L^−1^ [92], requiring a strong buffering mechanism [93]. Moreover, overexpression of low-affinity CAX transporters does not hinder normal growth [86], pointing to spatial compartmentalization, where [Ca^2+^]cyt Signaling is likely confined to specific microdomains. Specialized buffers may be physically separated from signaling proteins [93]; even if they release some free Ca^2+^, the ions are unlikely to be captured by signaling proteins and may be re-chelated. Thus, [Ca^2+^]cyt signaling may be characterized by spatially defined, transient Ca^2+^ spikes rather than slow global elevation and would not be disrupted by specialized cytosolic Ca^2+^ buffers.

The evolution of fast-acting, high-capacity Ca^2+^ buffers in the cytosol is plausible; for example, in mammalian retinal neurons, Ca^2+^-binding buffer proteins (CaB, CaR) chelate intracellular Ca^2+^ from the onset of light-evoked Ca^2+^ waves, truncating the transient response [111]. This provides a precedent for specialized, rapid Ca^2+^ buffers that intercept Ca^2+^ before it participates in signaling.

Therefore, the mechanism by which soluble Ca^2+^ complexes in the cytosol and vacuole indirectly lower cellular osmotic potential, thereby improving freezing tolerance—is likely more effective in calcicole ferns than in non-calcicole plants. A compelling example is *A. ruta-muraria* var. *schriesheimense*: although its laminae are thinner than the typical variety, it nonetheless exhibits a wintergreen habit [37].

#### 3.4.2. Physiological Hypothesis Two

The Ca-mediated osmotic adjustment pathway circumvents the need for organic solute synthesis and contributes to the freezing tolerance may operate to varying degrees among species, and we hypothesize that *Asplenium yishuiensis* uses soluble Ca^2+^ complexes more efficiently than its relatives.

*A. ruta-muraria* primarily relies on desiccation tolerance and its subcoriaceous laminae for freezing tolerance, and thus, even if it possesses specialized Ca-buffering proteins or chelators, they likely have relatively low Ca^2+^-binding capacity. Growing on nutrient-poor limestone substrates, it is unlikely that *A. yishuiensis* continuously synthesizes more specialized Ca-buffering proteins/chelators than its relative, even though these molecules themselves are soluble osmolytes. A more parsimonious hypothesis is that *A. yishuiensis* possesses specialized Ca-buffering proteins/chelators with higher per molecule Ca^2+^-binding capacity. Such molecules might be better termed specialized Ca^2+^-storage proteins/chelators.

Mg in plants is almost exclusively present as soluble Mg^2+^. As shown in Table 3, specimen 1 of *A. yishuiensis* has a Mg content of 7.69 g kg^−1^ DW, and specimens 2 and 3 also exceed the maximum uptake level of 4 g kg^−1^ DW reported for crops [7]. All three specimens contain Mg concentrations far above the critical toxicity limit for tea plant leaves (3.435 g kg^−1^ DW) and above the critical limits for dry banana leaves (3 g kg^−1^ DW) and coconut (2 g kg^−1^ DW) [7,12]. Given the role of Mg^2+^ in photosynthesis and membrane ionic currents, the free Mg^2+^ level in the cytosol must be strictly regulated. Fe content is also remarkably high. Mitra (2015) indicated that an Fe^2+^ concentration above 10 μM may cause toxicity and reduce growth parameters [7]. Under an extraction ratio of 1 g DW to 10 mL solution, a 10 μM Fe^2+^ solution corresponds to a minimum accumulation of 5.58 mg kg^−1^ DW; the actual value could be higher. However, the Fe content in *A. yishuiensis* fronds reaches 239 mg kg^−1^ DW. Nevertheless, no visible signs of Mg or Fe toxicity have been observed in *A. yishuiensis*.

This tolerance is likely attributable to specialized Ca^2+^-storage proteins in *A. yishuiensis*. Multiple studies have shown that, in many animals and plants, a wide range of metal ions—including Na^+^, Mg^2+^, Fe^2+^, Zn^2+^, Cu^2+^, and even K^+^—can be bound by CaBPs [7]. These cations compete with Ca^2+^ for binding, although some CaBPs do not exhibit competition between Ca^2+^ and other cations due to distinct binding sites. Moreover, certain CaBPs, after binding Ca^2+^, enhance their chelation capacity for other cations [112,113,114,115,116,117,118]. Chelated cations are no longer present as free ions, thereby avoiding toxicity that could arise from their excess. It has been hypothesized that the specialized Ca^2+^-storage proteins in *A. yishuiensis* are highly efficient, with each protein molecule capable of binding numerous Ca^2+^ ions. Consequently, their capacity to bind other cations is also strong, enabling the plant to absorb substantial amounts of trace elements, such as Fe^2+^. This mechanism may also explain the accumulation of V at concentrations up to 1.07 mg kg^−1^ DW from limestone soils naturally poor in V (lower than typical soils), remarkably exceeding the average value (0.502 mg kg^−1^ DW) reported for some medicinal plants [7]. Within plants, V is bio-transformed into V^4+^, which can similarly be chelated by specialized Ca^2+^-storage proteins.

However, although *A. yishuiensis* contains higher levels of Zn and Cu than *Nandina domestica* and *Sitobolium wilfordii*, these levels are not high compared with those in most plants. This observation suggests that the specialized Ca^2+^-storage proteins in *A. yishuiensis* possess binding sites specifically for Fe^2+^ and V^4+^, whereas other trace elements lack such dedicated sites. Additionally, CaBPs can shift the Fe redox equilibrium from Fe(III) to Fe(II) under aerobic conditions [114], a property that may enhance Fe bioavailability for human absorption.

Interestingly, in most plants, Mg content is lower than Ca content. In the three specimens of *N. domestica*, the highest Mg content reached 1.58 g kg^−1^ DW, which was far below the lowest Ca content of 2.89 g kg^−1^ DW. In contrast, *A. yishuiensis* accumulates Mg at levels comparable to or even substantially exceeding its Ca content (e.g., 7.69 g kg^−1^ Mg vs. 4.97 g kg^−1^ Ca). Even though Mg^2+^ may compete with Ca^2+^ for binding sites, the Ca concentration in limestone is significantly higher than that of Mg; therefore, Ca^2+^ would not be expected to lose the competition to Mg^2+^. Moreover, it is possible that Ca^2+^ and Mg^2+^ do not compete with each other for the specialized Ca^2+^-storage proteins but instead act cooperatively, allowing their combined content to reach as high as 12.66 g kg^−1^ DW. In summary, this phenomenon warrants further investigation.

Of course, *A. yishuiensis* does not absorb low amounts of Ca. Its Ca content is higher than that of *N. domestica* and meets the standard of typical calcicole plants. Although *S. wilfordii* also has a higher total Ca content than *N. domestica*, it is a widespread species that can survive away from limestone, suggesting that its soluble Ca^2+^ concentration is not high. While the total Ca content of *A. yishuiensis* does not reach the upper limit of calcicole ferns, its soluble Ca^2+^ concentration is likely higher than that of *N. domestica* and *S. wilfordii*, and perhaps even higher than that of many other calcicole ferns. This inference can be drawn from its K^+^ content. As mentioned above, rhizosphere Ca^2+^ can promote the release of NEK from limestone [11]. Exchangeable K^+^ in limestone is scarce, but NEK is relatively abundant. The release rate of NEK is largely dependent on the K^+^ concentration, and certain thresholds of K^+^ concentration prevent further NEK release from minerals. These K^+^ thresholds are related to mineral type and are increased in the presence of Ca^2+^ or Na^+^ in solution. Ca^2+^ or Na^+^ may increase soil K availability not only through their exchange effect on initial exchangeable K^+^, but also through their positive effect on the release of initial NEK by elevating the K^+^ concentration thresholds required for NEK release [11]. This indicates that the rhizosphere Ca^2+^ concentration of *A. yishuiensis* far exceeds that of *S. wilfordii*. As previously noted, cell-specific Ca^2+^ storage correlates with [Ca^2+^]apo and soil solution Ca^2+^ [90], with soluble Ca^2+^ playing the greatest role. The specialized Ca^2+^-storage proteins of *A. yishuiensis* may increase soluble Ca^2+^ within the plant, thereby raising the rhizosphere Ca^2+^ concentration and ultimately increasing the K^+^ concentration thresholds.

Furthermore, a low concentration of K^+^ in solution can inevitably facilitate NEK release from K-bearing minerals. The NEK in a mineral can only be exploited by plant roots when the minimum external K^+^ concentration at which K^+^ is taken up by the roots is lower than the K^+^ threshold for NEK release from that mineral. Consequently, the K^+^-uptake capacity of roots at low K^+^ concentration determines the plant’s NEK uptake capacity. In *A. yishuiensis*, the specialized Ca^2+^-storage proteins—even if they have a low affinity for K^+^—can still promote root K^+^ uptake, thereby lowering the rhizosphere K^+^ concentration and promoting NEK release from minerals.

Both factors indicate that *A. yishuiensis*, which possesses specialized Ca^2+^-storage proteins, can utilize substantial amounts of NEK, whereas *S. wilfordii* growing in the same limestone habitat cannot. The K content of *A. yishuiensis* (22.7 g kg^−1^ DW) is even 2.63-fold higher than that of the vigorously growing, cultivated *N. domestica* (8.62 g kg^−1^ DW).

All of the K, Ca, Mg, Fe, Cu, Zn, and V accumulated in *A. yishuiensis* provide positive charges that require substantial amounts of negative charges for balance. Consequently, *A. yishuiensis* takes up large quantities of inorganic anions, and its Cl, P, S, and N contents are significantly higher than those of *S. wilfordii* and *N. domestica*, which have comparable total Ca contents. Notably, *A. yishuiensis* not only accumulates high levels of Cl but also absorbs more than 2 g kg^−1^ P from the P-poor limestone soil. This value approaches the high end of the general plant range (0.05–0.5% DW [7]) and far exceeds the mean P content (0.146% DW) of terrestrial plant leaves in China [8]. These soluble cations—together with their accompanying inorganic anions—may substantially lower the osmotic potential under chronic AR stress. In summary, the Ca-mediated osmotic adjustment pathway enhances the freezing tolerance of *A. yishuiensis*.

This proposed mechanism does not preclude other freezing-tolerance strategies, such as exceptionally efficient supercooling capacity (e.g., specialized AFPs) and compatible osmolytes (primarily carbohydrates). However, the present discussion focuses specifically on the specialized osmotic adjustment pathway of *A. yishuiensis*.

##### Experimental Design to Test Whether Inorganic Anions Significantly Lower Cellular Osmotic Potential in *Asplenium yishuiensis*

Experiment 1: Direct charge measurement of native specialized Ca^2+^ storage proteins or chelators in *A. yishuiensis*

- Objective: Determine whether native specialized Ca^2+^ storage proteins or chelators from *A. yishuiensis* present a net charge-positive after Ca^2+^ loading.
- Approach: Isolate candidate Ca^2+^ storage proteins or chelators (e.g., via Ca^2+^-affinity chromatography) from *A. ruta-muraria* and *A. yishuiensis* grown under high-Ca^2+^ conditions (AR). Measure zeta potential and net charge by capillary electrophoresis before and after Ca^2+^ saturation.
- Prediction: Ca^2+^-storage proteins or chelators from *A. yishuiensis* grown under AR will exhibit a significant increase in positive charge after Ca^2+^ binding, confirming their capacity to attract multiple inorganic anions.

Experiment 2: Anion accumulation profiling

- Objective: Test whether high-Ca^2+^ stress induces significant accumulation of mobile anions in *A. yishuiensis*.
- Approach: Quantify cytosolic Cl^−^, NO_3_^−^, and malate^2−^ concentrations (using ion chromatography or fluorescent sensors) in *A. yishuiensis* and *Adiantum capillus-veneris* after 4 weeks of growth on calcareous soils.
- Prediction: *A. yishuiensis* will show significantly higher Cl^−^, NO_3_^−^, or malate^2−^ levels under AR, while the *Adiantum capillus-veneris* will not.

Experiment 3: Anion channel inhibition

- Objective: Establish causality between anion influx and osmotic adjustment.
- Approach: Treat high-Ca^2+^-grown *A. yishuiensis* with anion channel inhibitors (e.g., niflumic acid for Cl^−^ channels, bafilomycin for vacuolar malate transporters). Measure cytosolic osmolarity (via protoplast incipient plasmolysis) and freezing tolerance (LT_50_) after 7 days.
- Prediction: Anion channel inhibition will abolish the high-Ca^2+^-induced increase in osmolarity and freeze tolerance, confirming that anion influx is the causative agent.

In addition to the three experiments described above, the following experiments should be conducted in future research:

1. While the morphological and anatomical evidence robustly supports the recognition of *A. yishuiensis* as a distinct species, future studies incorporating population-level sampling and molecular phylogenetic analyses would provide additional resolution of its evolutionary relationships and genetic diversity
2. In addition to the calcium-mediated osmotic adjustment proposed here, other well-known cryoprotective mechanisms—such as the accumulation of compatible osmolytes, cold-regulated proteins, and antioxidant systems—may also contribute to the remarkable freezing tolerance of *A. yishuiensis*. Future studies should investigate these pathways to provide a more comprehensive understanding
3. Freezing tolerance (LT_50_) via controlled freezing assays and electrolyte leakage under a temperature gradient (0 °C, −5 °C, −10 °C, −20 °C).

##### Nitrate (NO_3_^−^) and Chloride (Cl^−^) Are the Most Effective Inorganic Anions for Lowering Cellular Osmotic Potential

The principal inorganic anions available in limestone substrates that can contribute to lowering cellular osmotic potential include NO_3_^−^, Cl^−^, SO_4_^2−^, H_2_PO_4_^−^/HPO_4_^2−^, and—under specific conditions—HCO_3_^−^. Among these, NO_3_^−^ and Cl^−^ are particularly effective osmotica. NO_3_^−^ also serves as a major nitrogen source [98,119]. SO_4_^2−^ is less effective and toxic at higher concentrations (Table 3 and Figure 3 reveal that the S content in *A. yishuiensis* is relatively low, with even lower levels observed in *N. domestica* and *S. wilfordii*); phosphate ions function primarily as nutrients; HCO_3_^−^, though abundant in alkaline calcareous soils, is rapidly metabolized in the cytoplasm and contributes to osmotic adjustment only after conversion to malate anions, a process energetically costlier than using NO_3_^−^ or Cl^−^ directly [100].

NO_3_^−^ is the chief vacuolar nitrogen store and can serve as an important osmoticum, with vacuolar concentrations ranging from 30 to 50 mmol L^−1^ (up to 300 mmol L^−1^ in some tissues) while cytosolic levels are strictly maintained at 4–5 mmol L^−1^ [120,121]; under salinity, it contributes significantly to osmotic adjustment, and its transport can be coordinated with Na^+^ movement [82]. Acquisition rates of NO_3_^−^ and K^+^ are often positively correlated, likely due to charge balance [122], and the same may hold for NO_3_^−^ and Ca^2+^.

However, NO_3_^−^ is metabolized and its use as an osmoticum is energetically wasteful compared to K^+^ [82,84]. Its uptake requires energy whereas Cl^−^ uptake does not [123]. Plants regulate total vacuolar anionic content—chiefly the sum of Cl^−^ and NO_3_^−^—so that uptake of the two is negatively correlated [83,124]. When NO_3_^−^ supply is limited, plants compensate by stimulating Cl^−^ absorption for the deficiency of negative charges [82,119]. Cl^−^ is a highly effective osmoregulatory molecule due to its high availability, mobility, low molecular weight, and metabolic inertness. It specifically stimulates plant cell osmolarity and turgor by (i) activating vacuolar V-type ATPase, thereby enhancing ion compartmentalization, and (ii) forming atypically stable solvation shells that favor water retention. Compared with anionic macronutrients (NO_3_^−^, SO_4_^2−^, PO_4_^3−^), Cl^−^ salts more effectively promote osmolarity, water content, relative water content, turgor, and leaf succulence [84]. The subsucculent laminae of *A. yishuiensis* (Figure 4) provide morphological evidence consistent with substantial Cl^−^ accumulation in this species, and further indicate that it accumulates more Cl^−^ than *A. ruta-muraria*.

Cl^−^ also specifically stimulates cell elongation [84], consistent with the larger mesophyll cells of *A. yishuiensis*. Therefore, Cl^−^ stimulation of larger cells with higher osmotic capacity and relative water content gives rise to plant tissues with superior water storage capacity [84]. As Jones and Gorham (2002) noted, “there is undoubtedly a close relationship between NaCl growth promotion and succulence [125]”—explaining the high water content of *A. yishuiensis* fronds.

Excessive Cl^−^ can induce oxidative stress [82], though a strong link between mesophyll Cl^−^ accumulation and reduced photosynthesis is not consistently found [85]. Plants accumulate Cl^−^ to macronutrient levels (15–50 mg g^−1^ DW), a range beneficial and non-toxic. Cl^−^ is preferentially compartmentalized in the vacuole. Cytosolic [Cl^−^] increases with external [Cl^−^] (6–360 mmol L^−1^; [124]), but harmful accumulation is limited to 30–50 mmol L^−1^ [125]. Thus, Cl^−^ is the dominant inorganic anion in plant cells, with leaf contents comparable to those of K^+^ (cytosolic K^+^ stably maintained at 100–200 mmol L^−1^; [78,125]. If vacuolar Cl^−^ reaches 200 mmol L^−1^, its theoretical osmotic contribution (excluding other anions) would be approximately −0.94 MPa—a substantial fraction of the total cellular osmotic potential (−1.0 to −2.5 MPa). Moreover, the greater abundance of soluble Ca^2+^ in calcicole ferns would attract proportionally more Cl^−^ into their cells.

Limestone substrates are inherently poor in nitrogen and chlorine. One might ask whether *A. ruta-muraria* primarily relies on its subcoriaceous laminae and DT for freezing tolerance simply because of limited external N and Cl^−^ availability—yet evidence suggests otherwise: populations on coastal limestone, where Cl^−^ is abundant, show no tendency toward subsucculent laminae [126]. This indicates that *A. ruta-muraria* lacks the specialized Ca^2+^-storage proteins/chelators of *A. yishuiensis*, and is therefore unable to accumulate sufficient Cl^−^. Thus, the specialized Ca-mediated osmotic adjustment pathway in *A. yishuiensis*—which circumvents energy-intensive organic solute synthesis—offers a more effective and energetically advantageous strategy than that of *A. ruta-muraria*, contributing substantially to its remarkable freezing tolerance.

### 3.5. Medicinal Properties and Future Perspectives

*A. ruta-muraria* has long been recognized as a medicinal herb in Europe, with historical records noting its use as an expectorant and aperitif [19,127], and modern studies confirming its applications as astringent, emmenagogue, and treatment for kidney stones [10,54]. As a closely related species, *A. yishuiensis* may possess similar medicinal potential; moreover, as discussed above, it also shows promise as an adjuvant therapy for diabetes mellitus and iron-deficiency anemia. This plant can absorb substantially higher amounts of V and relatively large amounts of Fe from barren limestone than ordinary plants. Therefore, under artificial cultivation with applied lime pellets or calcareous mortar, it is expected to accumulate even more V and Fe.

Furthermore, the genetic basis underlying the remarkable freezing tolerance of *A. yishuiensis* may serve as a valuable resource. Whether the genetic basis could be utilized to enhance stress resistance and pharmacological efficacy in related species, such as *A. ruta-muraria*, remains to be investigated in future studies.

### 3.6. Potential Applications in Enhancing Stress Tolerance of Crops

Since *A. yishuiensis* achieves freezing tolerance primarily through osmotic adjustment—by accumulating inorganic ions to lower cellular osmotic potential—this mechanism should confer tolerance to freezing, cold, and drought, as they are all fundamentally forms of water stress. This Ca-mediated osmotic adjustment pathway thus has broad applicability for coping with water-imbalance stresses, including salinity-alkalinity stress. Nieves-Cordones et al. (2019) [84] noted that rapid osmotic adjustment to water deficit results from fast vacuolar accumulation of inorganic ions (e.g., K^+^, Cl^−^), allowing rapid recovery of cell osmotic potential and turgor. Therefore, cellular mechanisms and agricultural practices that improve K^+^, Cl^−^, and water transport are potential targets for breeding drought-tolerant crops [84].

Whether the specialized Ca^2+^ storage proteins or chelators of *A. yishuiensis* could be utilized to enhance stress tolerance in crops remains to be investigated in future studies.

## 4. Materials and Methods

**Study site and plant material**—The new species, *Asplenium pekinense*, and fronds of *Sitobolium wilfordii* were collected from the Qianfo Hill scenic area. Leaves of *Nandina domestica* 1 were collected from the nursery greenhouse of Beijing Forestry University. Leaves of *N. domestica* 2, 3 were purchased from a flower market in Beijing. A single complete individual (Jue Wang & Qingchun Wang 20250820-1) of the new species was designated as the holotype. On 10 October 2025, additional materials were collected, including primarily fronds from multiple individuals and one complete specimen of *A. pekinense*. A subset of fronds, together with the entire *A. pekinense* plant, were sealed in plastic bags and kept for stereomicroscope observation; the rest were fixed in FAA solution and stored at 4 °C for two days prior to sectioning and staining. Additionally, 1 g of fresh fronds was collected, without fixation, pressed in a book, and air-dried naturally until May of the following year. This sample was designated as *A. yishuiensis* 1.

On 5 May 2026, additional frond samples of the new species were collected from the Qianfo Hill scenic area. Two groups of samples were obtained, each consisting of 1 g fresh weight of fronds from five distinct individuals, and no individual was shared between the two groups. The two samples were designated as *A. yishuiensis* 2 and *A. yishuiensis* 3. Then, the samples of *A. yishuiensis* 2, 3 and *N. domestica* 1, 2, 3 were dried at 70–80 °C for 48 h until a constant dry weight was achieved. Subsequently, the dried samples *A. yishuiensis* 1, 2, 3 and *N. domestica* 1, 2, 3 were sent to Hangzhou Yanqu Information Technology Co., Ltd., where the total concentrations of K, Ca, Mg, P, Fe, V, Cu, and Zn were determined using inductively coupled plasma mass spectrometry (ICP-MS) or inductively coupled plasma optical emission spectroscopy (ICP-OES).

Additionally, one compound frond was taken from each of *A. yishuiensis* 1, 2, and 3; one leaflet was taken from each of *N. domestica* 1, 2, and 3; and the lowest primary pinna was cut from the fronds of three distinct *S. wilfordii* individuals. A total of nine leaf samples were analyzed using an X-ray fluorescence (XRF) spectrometer (M4 Tornado plus; Bruker, Berlin, Germany).

**Morphological observation**—Morphological characters were observed and documented using an Evident SZ61 stereomicroscope (Evident Corporation, Tokyo, Japan) equipped with a digital camera (Evident Corporation, Tokyo, Japan). Observations focused on: (1) lamina (texture, marginal serration, venation); (2) petiole and rachises (sulcation, glandular hairs, other indumentum); (3) scales (morphology, distribution); and (4) fertile fronds, abaxially (sorus shape, indusium morphology); (5) density of glandular hairs.

**Anatomical observation**—After fixation, samples from petiole and lamina were rinsed in running tap water for 24 h and then dehydrated using an automatic tissue processor (KD-TS1, WRE2-120, Shanghai Jionglai Experimental Equipment Co., Ltd., Shanghai, China). Samples were then embedded in paraffin, sectioned at 12–15 μm with a rotary microtome (Leica 2235, Leica, Nussloch, Germany), stained with safranin O and fast green, mounted with neutral balsam (Bioss, Beijing, China), and observed/photographed under a Leica Versa 200 microscope (Leica Microsystems, Wetzlar, Germany).

During the preparation of this manuscript, the authors used deepseek for language refinement and grammatical corrections. The authors have reviewed and edited the output and take full responsibility for the content of this publication.

## 5. Conclusions

*Asplenium yishuiensis* is a new species from northern China, morphologically similar to traditionally medicinal fern *A. ruta-muraria*. The discovery of *A. yishuiensis* expands the diversity of the series *Asplenium* ser. *Variantia*. *A. yishuiensis* is distinguished by its herbaceous to subsucculent, sparsely glandular-hairy, and desiccation-sensitive laminae, in contrast to the coriaceous (to subcoriaceous), glabrous, and desiccation-tolerant laminae of *A. ruta-muraria*. Detailed examination further revealed consistent and distinct anatomical differences between *A. yishuiensis* and *A. ruta-muraria*.

Critically, *A. yishuiensis* lacks typical morphological and anatomical adaptations for freeze-tolerance (e.g., coriaceous texture, thick cuticle, small, multi-layered mesophyll cells, protective scales or a dense indumentum) common in temperate evergreen plants, and does not exhibit the poikilohydric strategy of a resurrection plant, revealing a previously unrecognized mechanism of freezing tolerance in ferns.

Based on detailed anatomical observations and physiological experiments, we propose a two-tiered physiological hypothesis for its winter-hardiness achieved in the absence of such traits: (1) Ca^2+^ itself has been recognized as an osmolyte. However, the accumulation of Ca^2+^ complexes (e.g., bound to soluble calcium-binding proteins) in the cytosol and vacuole may indirectly lower cellular osmotic potential in some plants, especially calcicole ferns. This is achieved by promoting K^+^ uptake and charge-driven inorganic anion uptake. As a result, inorganic ions become the primary osmotically active particles, which in turn improves freezing tolerance. (2) This mechanism may operate to varying degrees among species, and we hypothesize that *A. yishuiensis* uses Ca^2+^ complexes more efficiently than its relatives.

## Figures and Tables

**Table 1:** Mesophyll thickness in *Asplenium yishuiensis*.

Statistical Indicators	Group 1	Group 2
Sample size (n)	34	34
Mean mesophyll thickness	230.02 μm	235.58 μm
Standard deviation	26.47 μm	38.90 μm
Lower limit of 95% confidence intervals	220.78 μm	222.03 μm
Upper limit of 95% confidence intervals	239.26 μm	249.13 μm

**Table 2:** Summary of glandular hair area statistics.

Sample ID	Total Lamina Area (px^2^)	Total Glandular Hair Area (px^2^)	Proportion of Glandular Hair Area to Leaf Area (%)
S 1	543,200	780	0.14
S 2	1,056,800	1630	0.15
S 3	1,189,300	1750	0.15
S 4	1,245,700	2010	0.16
S 5	612,400	820	0.13
S 6	1,358,200	2130	0.16
S 7	925,600	1520	0.16
S 8	782,300	950	0.12
S 9	1,123,500	1870	0.17
mean	981,889	1496	0.15
standard deviation	267,321	492	0.02

**Table 3:** Elemental concentrations in fronds of *Asplenium yishuiensis* and *Nandina domestica*.

Sample ID	Test Item	Result	Unit
*Asplenium yishuiensis* 1	iron (Fe)	239	mg/kg
	calcium (Ca)	4.97 × 10^3^	mg/kg
	magnesium (Mg)	7.69 × 10^3^	mg/kg
	potassium (K)	1.85 × 10^4^	mg/kg
	phosphorus (P)	2.24 × 10^3^	mg/kg
	vanadium (V)	1.07	mg/kg
	sulfur (S)	2.27 × 10^3^	mg/kg
	copper (Cu)	5.62	mg/kg
	zinc (Zn)	30.6	mg/kg
*Asplenium yishuiensis* 2	iron (Fe)	80.5	mg/kg
	calcium (Ca)	4.26 × 10^3^	mg/kg
	magnesium (Mg)	4.39 × 10^3^	mg/kg
	potassium (K)	2.27 × 10^4^	mg/kg
	phosphorus (P)	2.28 × 10^3^	mg/kg
	vanadium (V)	0.334	mg/kg
	sulfur (S)	2.02 × 10^3^	mg/kg
	copper (Cu)	6.94	mg/kg
	zinc (Zn)	24.2	mg/kg
*Asplenium yishuiensis* 3	iron (Fe)	119	mg/kg
	calcium (Ca)	4.06 × 10^3^	mg/kg
	magnesium (Mg)	4.05 × 10^3^	mg/kg
	potassium (K)	1.95 × 10^4^	mg/kg
	phosphorus (P)	2.07 × 10^3^	mg/kg
	vanadium (V)	0.607	mg/kg
	sulfur (S)	2.03 × 10^3^	mg/kg
	copper (Cu)	5.71	mg/kg
	zinc (Zn)	22.5	mg/kg
*Nandina domestica* 1	iron (Fe)	28.1	mg/kg
	calcium (Ca)	2.89 × 10^3^	mg/kg
	magnesium (Mg)	1.58 × 10^3^	mg/kg
	potassium (K)	8.62 × 10^3^	mg/kg
	phosphorus (P)	1.58 × 10^3^	mg/kg
	sulfur (S)	1.35 × 10^3^	mg/kg
	copper (Cu)	3.41	mg/kg
	zinc (Zn)	11.4	mg/kg
*Nandina domestica* 2	iron (Fe)	79.4	mg/kg
	calcium (Ca)	5.26 × 10^3^	mg/kg
	magnesium (Mg)	1.29 × 10^3^	mg/kg
	potassium (K)	8.52 × 10^3^	mg/kg
	phosphorus (P)	2.11 × 10^3^	mg/kg
	sulfur (S)	1.56 × 10^3^	mg/kg
	copper (Cu)	7.65	mg/kg
	zinc (Zn)	14.4	mg/kg
*Nandina domestica* 3	iron (Fe)	47.8	mg/kg
	calcium (Ca)	5.41 × 10^3^	mg/kg
	magnesium (Mg)	1.03 × 10^3^	mg/kg
	potassium (K)	4.77 × 10^3^	mg/kg
	phosphorus (P)	1.25 × 10^3^	mg/kg
	sulfur (S)	1.18 × 10^3^	mg/kg
	copper (Cu)	4.72	mg/kg
	zinc (Zn)	10.6	mg/kg

## Data Availability

All data generated or analyzed during this study are included in this published article and its Supplementary Materials Files. Further inquiries can be directed to the corresponding author.

## Supplementary Materials

The following supporting information can be downloaded at: https://www.mdpi.com/article/10.3390/plants15121773/s1. Supplementary File S1: The raw measurement data of mesophyll thickness measurement.
