# Supplementary material for: Asplenium yishuiensis (Aspleniaceae), a New Wintergreen and Medicinal Fern from Northern China, Achieves Freezing Tolerance via a Calcium-Mediated Osmotic Adjustment Pathway
Source: Plants (Basel). 2026 Jun 8;15(12):1773. doi: 10.3390/plants15121773 (PMC13306970; doi:10.3390/plants15121773)
Supplement: Supplementary file 1 [file plants-15-01773-s001.zip › plants-4282015-supplementary.pdf]

The raw measurement data of mesophyll thickness measurement		
S1-1 pinnule 1	232.42 μm	235.01 μm
S1-1 pinnule 2	249.27 μm	240.30 μm
S1-2 pinnule 1	190.28 μm	191.12 μm
S1-2 pinnule 2	218.01 μm	232.12 μm
S2-1 pinnule 1	291.50 μm	285.54 μm
S2-1 pinnule 2	273.19 μm	273.51 μm
S2-2 pinnule 1	293.74 μm	290.03 μm
S2-2 pinnule 2	286.66 μm	279.04 μm
S3-1 pinnule 1	200.54 μm	207.41 μm
S3-1 pinnule 2	138.01 μm	143.50 μm
S3-2 pinnule 1	217.01 μm	198.65 μm
S3-2 pinnule 2	219.06 μm	226.08 μm
S4-1 pinnule 1	191.82 μm	194.25 μm
S4-1 pinnule 2	194.03 μm	199.00 μm
S4-2 pinnule 1	209.54 μm	202.80 μm
S4-2 pinnule 2	263.30 μm	272.16 μm
S5-1 pinnule 1	214.15 μm	224.76 μm
S5-1 pinnule 2	186.82 μm	216.42 μm
S5-2 pinnule 1	221.55 μm	237.10 μm
S5-2 pinnule 2	260.95 μm	267.05 μm
S6-1 pinnule 1	247.27 μm	296.59 μm
S6-1 pinnule 2	250.12 μm	264.50 μm
S6-2 pinnule 1	285.59 μm	260.70 μm
S6-2 pinnule 2	268.72 μm	243.51 μm
S7-1 pinnule 1	244.50 μm	239.05 μm
S7-1 pinnule 2	227.19 μm	182.99 μm
S7-2 pinnule 1	255.36 μm	225.08 μm
S7-2 pinnule 2	238.06 μm	224.01 μm
S8-1 pinnule 1	318.23 μm	311.63 μm
S8-1 pinnule 2	272.35 μm	334.30 μm
S8-2 pinnule 1	171.46 μm	183.80 μm
S8-2 pinnule 2	219.04 μm	224.16 μm
S9 pinnule 1	215.51 μm	221.82 μm
S9 pinnule 2	257.05 μm	238.00 μm
